# Transcriptomic and metabolomic profiling of strawberry during postharvest cooling and heat storage

**DOI:** 10.3389/fpls.2022.1009747

**Published:** 2022-10-12

**Authors:** Ting Zheng, Jinhua Lv, Ehsan Sadeghnezhad, Jianhui Cheng, Haifeng Jia

**Affiliations:** ^1^ Institute of Horticulture, Zhejiang Academy of Agricultural Sciences, Hangzhou, China; ^2^ College of Horticulture, Nanjing Agricultural University, Nanjing, China

**Keywords:** postharvest strawberry, cold stress, heat stress, transcriptome and metabolome, quality

## Abstract

Temperature is one of the most important factors regarding fruit postharvest, however its effects in the strawberry fruits quality in postharvest remains to be evaluated. In this study, the effects of cold and heat storage temperature on fruit quality of ‘Benihoppe’ strawberry were performed. The results showed that different temperatures could affect the metabolism of hormone, anthocyanin, reactive oxygen species (ROS), and transcription level of responsive factors. The synthesis of terpenoids, amino acids, and phenylpropanoids in strawberries were also changed under different temperatures, which finally changed the quality characteristics of the fruit. We found *HSF20* (*YZ1*)-overexpressed fruits were sensitive to cold and heat conditions but CBF/NF-Y (YZ9)-overexpressed fruits promoted coloring under cold treatment. This study clarified the effect of postharvest cooling and heat treatments on quality and transcriptional mechanism of strawberries fruits. Moreover, these results provided an experimental basis for further research on improving the quality of strawberry berries during postharvest periods.

## Introduction

Strawberry (*Fragaria ananassa* Duch.) as a fruit appreciated in the food and drug industry is noteworthy for its attractive color, unique flavor, and nutritional benefit (Zhang et al., 2019). Among different strawberry cultivars, ‘Benihoppe’ strawberry is very popular among consumers and it has become one of the most important varieties in China (Liu et al., 2016). ‘Benihoppe’ strawberry cultivar is considered for its perfect balance of sweetness and sourness that obtained by the hybridization of ‘Akihime’ (sweetness) and ‘Sachinoka’ (acidity) (Kim et al., 2015). It is a fruit with high nutritive value, which contains sugar, acid, and several proteins, as well as a good source of trace minerals (Pott et al., 2020).

Postharvest diseases decline the quality of harvested fruits and lead to short storage life in the market (Shin et al., 2007; Hashmi et al., 2013). Usually, strawberries can only be stored for 2-3 days under room temperature conditions, therefore, we need to consider the different strategies to protect fresh strawberries quality during storage. In order to maintain the commercial strawberry production during a postharvest time, there are the commonly used fresh-keeping technologies including low-temperature refrigeration and treatment with low-pressure, heat, UV-C, ozone, and calcium (Chen et al., 2011; Hashmi et al., 2013; Xu et al., 2019). Among them, low-temperature storage is the most common method of preservation and could effectively reduce their protein decomposition and the rate of decay, preserve the content of sugar and acidity, and inhibit the loss of water, which lead to the maintenance of the nutritional value and quality of strawberries (Ayala-Zavala et al., 2004; Cordenunsi et al., 2005). In addition, high ambient temperature hiked anthocyanin accumulation in postharvest strawberries through up-regulations of anthocyanin biosynthetic genes and transportation genes and also led to anthocyanin degradation by laccase genes expression at the same time, resulting in the fruits discoloration of postharvest strawberries (Peng et al., 2017; Zhang et al., 2019). Changes of physiological and molecular occurred in plant in response to low or high temperature have been extensively studied (Kaplan et al., 2004; Shin et al., 2007). Soluble sugars, amino acids, organic acids, hormones, and anthocyanins are important substances, which played crucial role in the resistance of plants to environmental changes (Zhang et al., 2011; Carmona et al., 2017; Jin et al., 2017). Heat shock proteins (HSPs) and AP2 family transcription factors CBFs are also important for plant to response to temperature stress (Chinnusamy et al., 2007; Zhu, 2016).

The metabolites for aroma and taste synthesis can be powerful tools for tracking the deterioration of strawberry under temperature changes during postharvest storage. The gene regulatory network in response to cold stress as well as multiple regulated metabolic networks have been studied in different species of plant (Cook et al., 2004; Kaplan et al., 2004; Lei et al., 2014; Maruyama et al., 2014; Bai et al., 2015; Zhang et al., 2019). Therefore, the association between the gene and metabolite networks is incredible and remains to be elucidated under temperature treatments through the identification of molecular mechanisms and biochemical pathways. Especially in strawberries, which the physical damage and pathogen attack could decrease the fruit quality in commercial storage conditions, has not been thoroughly studied. The increased ease and efficiency of RNA sequencing (RNA-Seq) tools will facilitate the study of the mechanisms underlying metabolite variation. On the basis of metabolite analysis, a stringent logical filter for high-throughput approaches could be set up and used to identify the relevant factors (Lou et al., 2014). The combined analysis of transcriptomic and metabolomics can provide a large amount of data on molecular and metabolic events under different storage conditions, which help to further analyze the metabolic process of harvested strawberry fruit (El-Sharkawy et al., 2015).

In this study, we investigated the transcriptomic and metabolic changes of ‘Benihoppe’ strawberry fruits during postharvest cooling and heat storage and clarify the response of strawberry endogenous metabolism to different temperatures. Our results provide experimental basis for further research to effectively preserve softening during postharvest storage and the quality of strawberry berries.

## Materials and methods

### Plant materials and treatments

**Figure 1:**
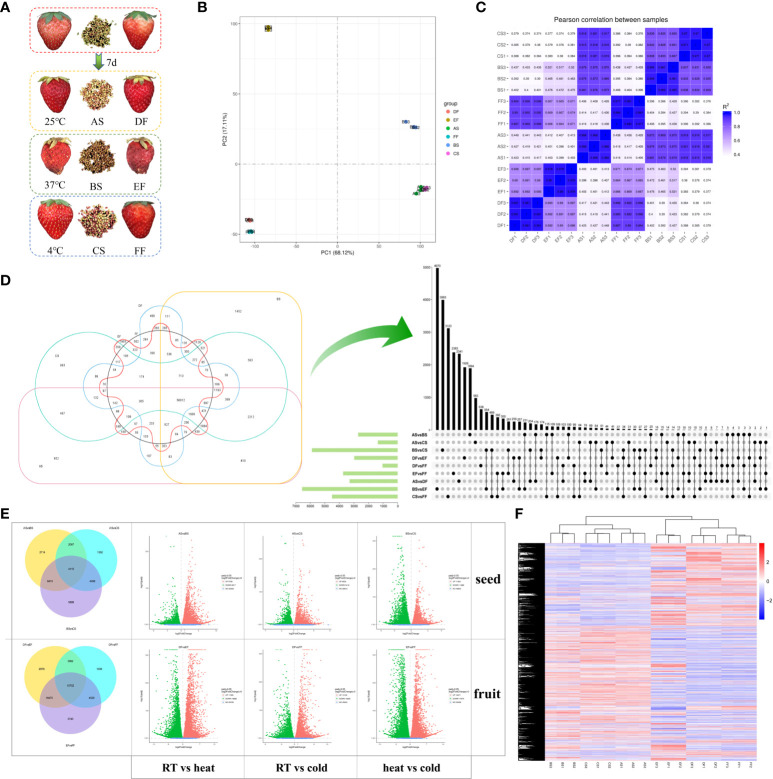
Differentially expressed genes (DEGs) in seeds and fruits of strawberries after 7 days under different storage temperature modes. **(A)** Strawberry fruits and seeds under different storage temperatures; **(B)** Principal component analysis (PCA) scatter plot of different samples based on the transcriptomic profiles; **(C)** Correlation coefficient graph of all samples; **(D)** DEGs in samples treated at cold, RT, and heat treatments. The 6-element Venn diagram was used to represent the number of genes with no difference in expression between different groups. The Upset diagram was used to more closely represented the intersection between different temperatures and samples. The green bar graph represents the size of each combination, while the black dot represents yes, the gray dot represents none, and the black bar graph represents the number of intersections. **(E)** Volcano map for the significance level of the DEGs. The horizontal axis represents the fold change of DEGs, and the vertical axis represents the significance level of the difference. Padj indicates the corrected p value after the multiple hypothesis test, the same as below. **(F)** Comparison of the transcription level of DEGs using heatmap analysis. Samples were divided into two groups including seeds (RT (AS), heat (BS), and cold storage (CS)) and fruits (RT (DF), heat (EF), and cold storage (FF)).

The strawberry fruits ‘Benihoppe’ (*Fragaria ananassa* Duch.) were collected from the vineyard of Nanjing Agricultural University (31°36′N, 119°10′E) at April in Nanjing, Jiangsu Province, China. All selected fruits were mature with uniform fruit size and intact shape, grown in a greenhouse under standard cultivation conditions (20°C-25°C, relative humidity of 70%-85%, 14 h/10 h light/dark cycles) during spring seasons. The harvested strawberries (28 days after anthesis) were placed in a transparent airtight box and treated with different temperatures including room temperature (RT, 25°C), low temperature (cold, 4°C), and high temperature (heat, 37°C), with a relative humidity of 90% in a constant temperature incubator. There are three repetitions for each treatment, and twelve fruits for each repetition. The samples were randomly collected after 7 days, and separated into two groups including seeds (at room temperature storage (AS), high temperature storage (BS), and low temperature storage (CS)) and fruits (berries under room temperature storage (DF), high temperature storage (EF), and low temperature storage (FF)) (**Figure 1A**), and immediately frozen in liquid nitrogen. All samples were stored at –80°C for subsequent analysis.

### Transcriptomic analysis

Total RNAs were extracted using a CTAB method according to the method of Wang et al. (2014). The RNA purity and integrity were assessed based on the A260/A280 absorbance ratio and 1.0% agarose gel electrophoresis. Library preparation and transcriptome sequencing were completed by the Beijing Novogene Technology Corporation (Beijing, China). All treatments were performed with three biological replicates. Differentially expressed genes (DEGs) analysis was performed using the DESeq R package (1.18.0) (Benjamini and Hochberg, 1995). Genes with |log2foldchange|>0 an the adjusted *p*-value< 0.05 were considered as differentially expressed (Zhang et al., 2020; Zhang et al., 2021; Zhang et al., 2021). The GO enrichment analysis and the statistical enrichment of DEGs in the KEGG pathway of DEGs were performed with the GOseq R software package and KOBAS software, respectively (Zhang et al., 2020).

### Transient expression levels of key genes

Using screening of transcriptome data, we selected six genes in response to temperature changes including: Hsp20/alpha crystallin family (XM_004307545, YZ1), Universal stress protein family (XM_004300412.2, YZ4), Senescence regulator (XM_004295368.2, YZ5), EF-hand domain pair (XM_004306435.2, YZ8), Histone-like transcription factor (CBF/NF-Y) and archaeal histone (XM_004291519.2, YZ9), and Protein phosphatase 2C (XM_004302777.2, YZ10) (**Table S1**). Full-length coding sequences of these genes were cloned from the cDNA of ‘Benihoppe’ strawberries. The cloned sequences were then transferred into the *pCAMBIA1302* vector to produce the transformed plasmids including *YZ1-GFP, YZ4-GFP, YZ5-GFP, YZ8-GFP, YZ9-GFP*, and *YZ10-GFP*. According to Zheng et al. (2020), the plasmids were subsequently transfected with Agrobacterium (strain *EHA105*) and used for the infection of the strawberries during their large green fruit period. For control (CK), samples were infected with the *pCAMBIA1302* vector alone. All treatments were performed with three biological replicates, and ten fruits for each repetition. The transformed strawberries were placed in incubators with 4°C, 25°C, 37°C. The samples were collected after 5 days, while the skin and seeds were removed, and the remaining fruit were used for subsequent analysis.

### Determination of physiological and biochemical parameters

The anthocyanin content was determined using spectrophotometry according to the method described by Zheng et al. (2020). Total anthocyanin was extracted using a methanol–HCl method. Samples (0.1 g) were soaked and incubated overnight in 5 mL methanol containing 0.1% (v/v) HCl in the dark at room temperature. Anthocyanin contents in samples were measured using the PH differential method. The absorbance of sample extracts at 520 and 700 nm was measured using a UV-2550 spectrophotometer (Shimadzu). The sugar and organic acid contents were determined using high-performance liquid chromatography (HPLC) according to the method of Zheng et al. (2020). 0.5 g samples were ground in liquid nitrogen and mixed with 1.5ml 80% ethanol, 85°C for 30 min, and centrifuge at 12000 g for 10 minutes. Aspirate the supernatant and repeat the above step. Mix the supernatant together and freeze-dry. 15 ml ultrapure water was used to dissolve lyophilized precipitate and filtered the solution with 0.22 water-filter for the measurement of soluble sugars and organic acids. The contents of hemicellulose, pectin, and cellulose were determined using related kits (Solarbio Life Sciences, Beijing, China). The enzyme activities of superoxide dismutase (SOD) and peroxidase (POD) were determined using enzyme activity test kit (Solarbio Life Sciences, Beijing, China). All experiments were performed three replicates.

The aroma components were determined using gas chromatography-mass spectrometry (GC-MS) (Zheng et al., 2020; Zheng et al., 2021). Samples (2 g) were ground and transferred to a headspace bottle (15 mL). Three millilitres of saturated NaCl solution were added to the samples, and 32.84 ng 3-nonanone was added as an internal standard. GC–MS analysis was performed automatically using a TSQ™ 9000 Triple Quadrupole GC–MS/MS System (Thermo Scientific, Nanjing, China) with a 50/30 μm DVB/CAR/PDMS Fibre (Supelco, USA) that was maintained at 40°C for 30 min. The MS conditions were as follows: EI mode: voltage, 70 eV; ion source temperature, 230°C; scanning rate, 2.88 scan·s^−1^; mass spectrometry detection range, 29–540 m·z^−1^; carrier gas, helium; flow rate, 1.0 mL min^−1^. The column temperature was programmed as follows: the initial temperature was set at 50°C for 6 min and then increased to 250°C at 6°C min^−1^, which was held for 3 min. The chromatographic and spectral data were evaluated using the TraceFinder software (Thermo Scientific, USA).

### qRT-PCR Analysis

The expression levels of genes involved in cell wall, anthocyanin and aroma accumulation were assayed in strawberries. cDNA was synthesized using a HifairII^®^ 1st Strand cDNA Synthesis SuperMix for qPCR (Yeasen, Shanghai, China). The qRT-PCR reactions consisted of 5 μL SYBR Premix Ex Taq™ (Takara, Japan), 0.3 μL of each primer (10 μM), 2 μL cDNA, and 2.4 μL RNase-free water in a total volume of 10 μL. The reaction was performed using a LightCycler 1.5 instrument (Roche, Germany), with the preliminary step at 95°C for 30 s followed by 35 cycles at 95°C for 5 s and 58°C for 35 s. The relative gene expression was calculated using the 2^–△Ct^ method (Zheng et al., 2021). For the verification of transcriptomic data, we also screened out 10 differentially expressed genes using qRT-PCR. Specific primers used for qRT-PCR are listed in **Table S2**.

### Metabolomic profile detection and analysis

Tissues (100 mg) were individually grounded with liquid nitrogen and the homogenate was resuspended with prechilled 80% methanol and 0.1% formic acid by well vortexing. The samples were incubated on ice for 5 min and then were centrifuged at 15000 rpm, 4°C for 5 min. A some of supernatant was diluted to final concentration containing 60% methanol by LC-MS grade water. The samples were subsequently transferred to a fresh Eppendorf tube with 0.22 μm filter and then were centrifuged at 15000 g, 4°C for 10 min. Finally, the filtrate was injected into the LC-MS/MS system analysis.

LC-MS/MS analyses were performed using a Vanquish UHPLC system (Thermo Fisher) coupled with an Orbitrap Q Exactive HF-X mass spectrometer (Thermo Fisher). Samples were injected onto an Hyperil Gold column (100×2.1 mm, 1.9μm) using a 16-min linear gradient at a flow rate of 0.2 mL/min. The eluents for the positive polarity mode were eluent A (0.1% FA in Water) and eluent B (Methanol). The eluents for the negative polarity mode were eluent A (5 mM ammonium acetate, pH 9.0) and eluent B (Methanol). The solvent gradient was set as follows: 2% B, 1.5 min; 2-100% B, 12.0 min; 100% B, 14.0 min; 100-2% B, 14.1 min; 2% B, 16 min. Q Exactive HF-X mass spectrometer was operated in positive/negative polarity mode with spray voltage of 3.2 kV, capillary temperature of 320°C, sheath gas flow rate of 35 arb and aux gas flow rate of 10 arb (Zhang et al., 2020).

For data analysis, we used Compound Finder 3.0 (CD 3.0, Thermo Fisher) for the processing of the raw data files generated by UHPLC-MS/MS and performed peak alignment, peak selection, and quantification for each metabolite. The main parameters were set as follows: retention time tolerance, 0.2 minutes; actual mass tolerance, 5ppm; signal intensity tolerance, 30%; signal/noise ratio, 3; and minimum intensity,100000. After that, peak intensities were normalized to the total spectral intensity. The normalized data was used to predict the molecular formula based on additive ions, molecular ion peaks and fragment ions. And then peaks were matched with the mzCloud (https://www.mzcloud.org/) and ChemSpider (http://www.chemspider.com/) database to obtained the accurate qualitative results. The peak area was used for quantitative analysis. The quality control (QC) sample was set to evaluate the system stability during the experiment, and the blank sample was used to remove background ions. The statistical software R (R version R-3.4.3), Python (Python 2.7.6 version), and CentOS (CentOS release 6.6) were used for statistical analysis. The screening of the differentially accumulated metabolites between different samples mainly dependent on VIP (Variable Importance in the Projection), FC (Fold Change) and P-value. In this experiment, the threshold was set as VIP *>* 1.0, FC *>* 1.5 or FC< 0.667 and P value< 0.05 (Zhang et al., 2021).

### Statistical analysis

Samples were analyzed using statistical analysis of variance (ANOVA) SPSS statistics 17.0 (SPSS Inc, Chicago, ILL, USA), TBtools v1.072 (Chen et al., 2020), and Origin Pro 9 (Origin Inc., Northampton, MA, USA). All experiments were performed at least three replicates. For the principal component analysis (PCA) and the heatmap analysis, we used the normalized transcriptome data after taking the logarithm (LogFPKM) and the metabolites content data. MapMan (version 3.6.0RC1, Berlin, Germany) was used to exhibit the differences in the expression of genes involved in various functional modules.

## Results

### Postharvest cooling and heat changed transcriptome profile in strawberries

To determine the molecular mechanism of temperature on strawberry, we performed RNA-Seq on seeds (S) and fruits (F) under three different temperatures (A and D for 25°C; B and E for 37°C; C and F for 4°C) (**Figure 1A**). Sequencing of the samples generated about 8.25 G data and 55,012,379 clean reads for each sample (**Table S3**). Compared with the strawberry genome database, the mapping rate of the samples was 91.17%, fully reflecting the changes in the transcription level of strawberries under different treatment conditions. Meanwhile, 2416 new genes were identified in this study (**Table S4**), which may be employed in further functional research.

According to the PCA analysis of transcriptome data, we observed a strong correlation between cold (4°C) and RT (25°C) treatments in seeds (AS/CS) and fruits (DF/FF) than heat (37°C) and RT treatments in both (**Figures 1B, C**). It can be seen from the scattered points distribution that the scores of fruits and seeds change regularly under different temperature modes. The distance between fruit and seed samples is very large, especially in the direction of PC1, indicating that PC1 is closely related to the type of samples including seeds and fruits. The distance between heat-treated strawberry and other samples is very long in the direction of the PC2 axis, indicating that PC2 is related to temperature differences. These results indicated that strawberries have a greater difference in response to heat and cold treatments. The changes in the transcriptome level caused by cold stress are greater than heat, and this phenomenon is more obvious in seeds than fruits.

To verify the reliability of the RNA-seq results, we randomly selected 10 DEGs for qRT-PCR. Results showed that the majority of genes were in a similar expression pattern with those of the sequencing results (**Figure S1**). Temperature treatments induced a difference in the number of DEGs (**Figures 1D, E**). In seeds and fruits, heat treatment brought more DEGs than postharvest cooling. The DEGs in fruits were ranged from 56.6 - 96.9% that was higher than that in seeds under the same temperature condition. The number of up and down DEGs were almost the same, ranged from 45% to 55%. We observed the largest number of DEGs in EFvsFF class, while ASvsCS was the smallest. Meanwhile, the transcription levels of DEGs between different groups were presented in the cluster heat map (**Figure 1F**). The groups under cold and RT conditions were divided into one category, suggesting that the responses of the strawberry to different temperatures have both common characteristics and specific properties.

### GO enrichment and KEGG pathway analysis of DEGs

Postharvest heat treatment of fruits led to the 18,682 and 17,365 up- and down-regulated genes, respectively compared to RT (EFvsDF). These genes were significantly enriched in biological processes (BP), cellular components (CC), and molecular functions (MF). The three most abundant of BP are included protein localization, establishment of protein localization, and cellular catabolic process. Among the 30 GOs that were significantly enriched, the number of genes enriched in each GO in DF was more than that in EF except for phosphorelay sensor kinase activity. Compared to RT-treated fruits, the cold-treated fruits (FFvsDF) had 10,025 and 10,136 up- and down-regulated genes, respectively. These genes were significantly enriched in the BP and MF, which means cold does not bring significant changes in CC. The BP with the largest number of enriched genes was the single-organism carbohydrate metabolic process, and the MF is oxidoreductase activity, acting on CH-OH group of donors. Similarly, the number of enriched genes in each GO belonged to DF was more than that in FF (**Figures 1E**, **S2**).

Compared to RT treatment, heat-treated seeds (BSvsAS) had 8,517 and 9,789 up- and down-regulated genes, respectively. These genes were significantly enriched in BP, CC, and MF. Single-organism carbohydrate metabolic process and nucleobase-containing small molecule metabolic process were the two most significant GOs related to BP, while active transmembrane transporter activity and GTPase activity were the two most significant GOs related to MF. GOs related to BP and CC showed a higher expression level in heat than that in RT. Only few CC-related genes, such as proton-transporting ATP synthase complex, had higher expression levels in heat-treated seeds than that in RT. While the cold-treated seeds (CSvsAS) had 5,410 and 6,632 up- and down-regulated genes, showing a completely different pattern from heat-treated seeds. The GOs related to CC were only enriched in chromatin, nucleosome, lipid particle, monolayer-surrounded lipid storage body, protein-DNA complex, and DNA packaging complex, and were up-regulated under cold conditions. BP and MF were mainly enriched in the cellular carbohydrate metabolic process, cellular carbohydrate metabolic process, and oxidoreductase activity, acting on CH-OH group of donors (**Figures 1E**, **S2**).

**Figure 2:**
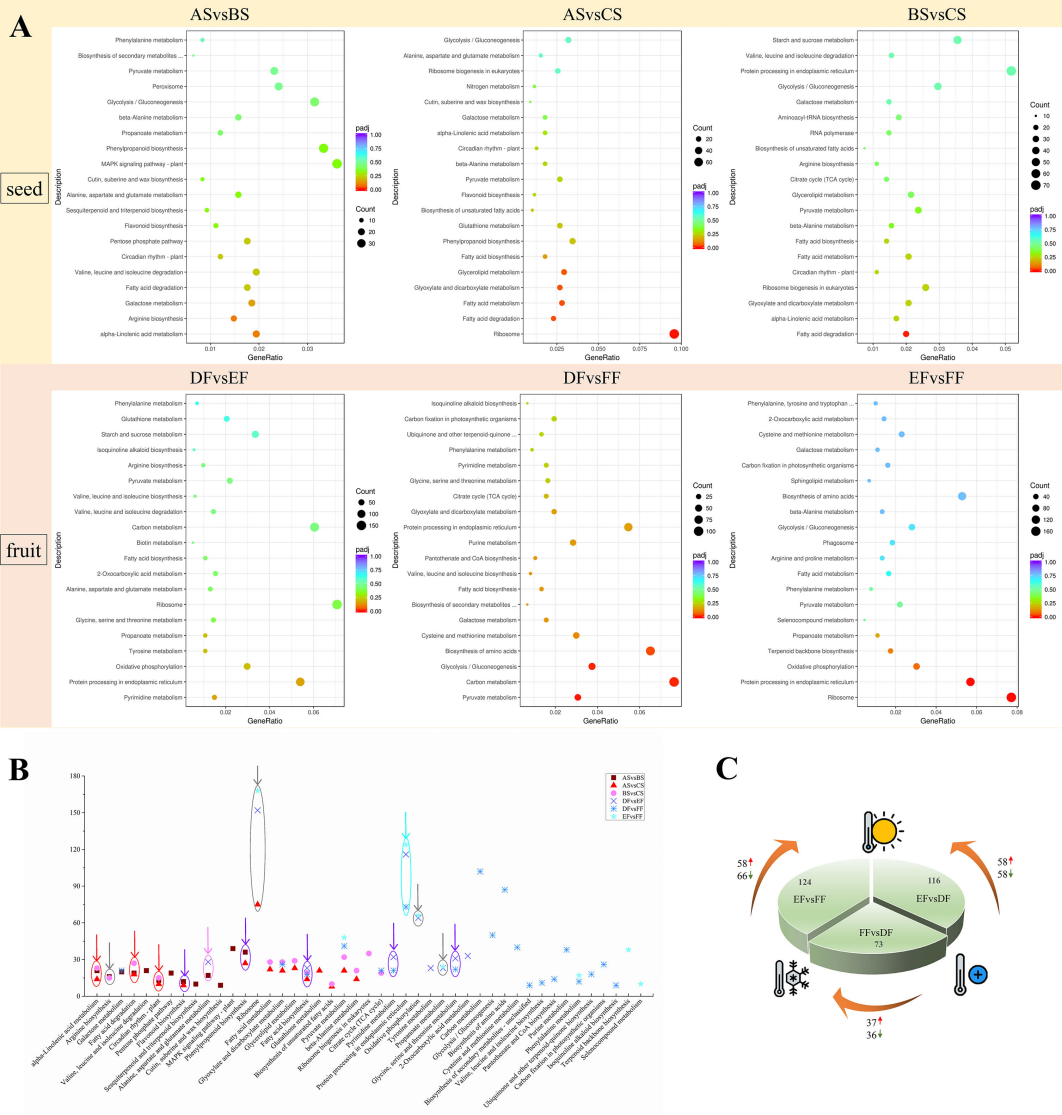
The Kyoto Encyclopedia of Genes and Genomes (KEGG) pathways analysis with significant enrichment of DEGs in seeds and fruits of strawberry. **(A)** KEGG pathways were enriched with up-regulated DEGs between different groups. GeneRatio represents the ratio of the number of differential genes annotated to the KEGG pathway to the total number of DEGs. Count represents the number of DEGs annotated to the KEGG pathway. padj represents the p-value corrected by multiple hypothesis testing. **(B)** Analysis of significant KEGG pathway enriched between different groups (ASvsBS, ASvsCS, BSvsCS, DFvsEF, DFvsFF, and EFvsFF). The red arrow represents the simultaneous enrichment in ASvsBS, ASvsCS, and BSvsCS, the gray arrow represents the simultaneous enrichment in RT vs Heat and Heat vs Cold (ASvsBS and BSvsCS; DFvsEF and EFvsFF), the purple arrow represents the simultaneous enrichment in RT vs Heat and RT vs Cold (ASvsBS and ASvsCS; DFvsEF and DFvsFF), the pink arrow represents the simultaneous enrichment in RT vs Heat (ASvsBS and DFvsEF), and the cyan arrow represents the simultaneous enrichment in DFvsEF, DFvsFF, and EFvsFF. **(C)** Analysis of the protein processing in endoplasmic reticulum pathway of fruits under different temperature treatments.

Using KEGG pathway analysis, we counted the number of paths for DEGs for ASvsBS, ASvsCS, BSvsCS, DFvsEF, DFvsFF, and EFvsFF, which were 13, 14, 13, 10, 20, 8 pathways (p-value< 0.05), respectively (**Figure 2A**). In treated-seeds with different temperatures, alpha-linolenic acid metabolism, fatty acid degradation, and circadian rhythm were highlighted, while protein processing in endoplasmic reticulum indicated a significant difference in berries. This means seeds and fruits possess the different mechanisms in response to different temperatures (**Figure 2B**). According to the analysis of protein processing in endoplasmic reticulum pathway of fruits, there were 58 up-regulated and 58 down-regulated DEGs related to shifting of RT to heat, 37 and 36 up- and down-regulated DEGs, respectively for shifting from RT to cold, 58 and 66 up- and down-regulated DEGs, respectively for shifting from cold to heat treatment (**Figure 2C**), showing that the berry will mobilize a large number of genes to deal with heat temperature.

Seeds and fruits behave differently between cold and heat treatments compared to RT. The seeds mainly involved flavonoid biosynthesis and phenylpropanoid biosynthesis, and the fruits are mainly reflected in fatty acid biosynthesis, pyrimidine metabolism and glycine, serine and threonine metabolism. Seeds and berries also have a common pathway in response to heat treatment including; alanine, aspartate and glutamate metabolism.

### Multiple stress pathways responding to heat and cold treatments

In response to high- and low-temperature treatments, profile of DEGs in berries changed more obviously in transcriptome level than seeds (**Figure S3**). Compared to RT, the transcription level of most genes in heat treatment was up-regulated, which belonged to biotic and heat stresses, but suppressed in cold treatment (**Figures S3A, C**). This also suggests the interaction between heat and biotic stresses that makes strawberries more susceptible to pathogens under postharvest heat, however, the genes involved in drought, salt, touch, wounding, and light pathways were less affected. The cold-response genes were only partially upregulated while other abiotic stresses did not change more under storage temperature (4°C), which is commonly used in daily life (**Figures S3B, D**). In redox reactions, genes belonged to Ascorbate-Glutathione pathway severely affected under temperature changes. Meanwhile, postharvest heat treatment altered the expression of genes involved in cell division and cell cycle more than cold treatment. On the whole, heat and cold treatments affected DEGs less in seeds than fruits, except for genes involved in biotic- and heat-responsive mechanisms. In addition, both heat and cold treatments have greatly affected the transcription of most genes in the hormone biosynthesis pathway. Heat treatment also up-regulated the most genes involved in the biosynthesis pathway of benzyl adenine (BA), cytokinin (Cyt), salicylic acid (SA), gibberellin (GA), and some genes in the pathway of IAA and ethylene (Eth). Meanwhile, cold treatment caused the down-regulation of some genes in the biosynthesis pathways of Eth and jasmonate (JA).

After postharvest cooling and heat treatments, the expression levels of various transcription factor family members were differentially changed in berries and seeds (**Figure S4**). In strawberry fruit, heat treatment affected the transcription level of genes belonged to almost all transcription factor family, especially AP2-EREBP, bHLH, bZIP, C2H2, HB, HSF, MYB, NAC, WRKY, AS2, AuxIAA, Histone, GRAS, and SET-domain, although histone family members were down-regulated under heat conditions (**Figures S4A, C**). Under low-temperature conditions, transcription factors show different changes (**Figures S4B, D**). Many transcription factor families were not affected by cold treatment, such as HSF, histone, and SET-domain but were severely affected by heat treatment. Similar to heat treatment, bHLH, MYB, WRKY, and AS2 were also transcription factor families that significantly affected by cold treatment, indicating that these genes may play an important role in signal transduction after heat and cold treatments. In seeds, transcription factors showed a relatively consistent pattern but not significant trend in expression, especially up-regulated genes.

**Figure 3:**
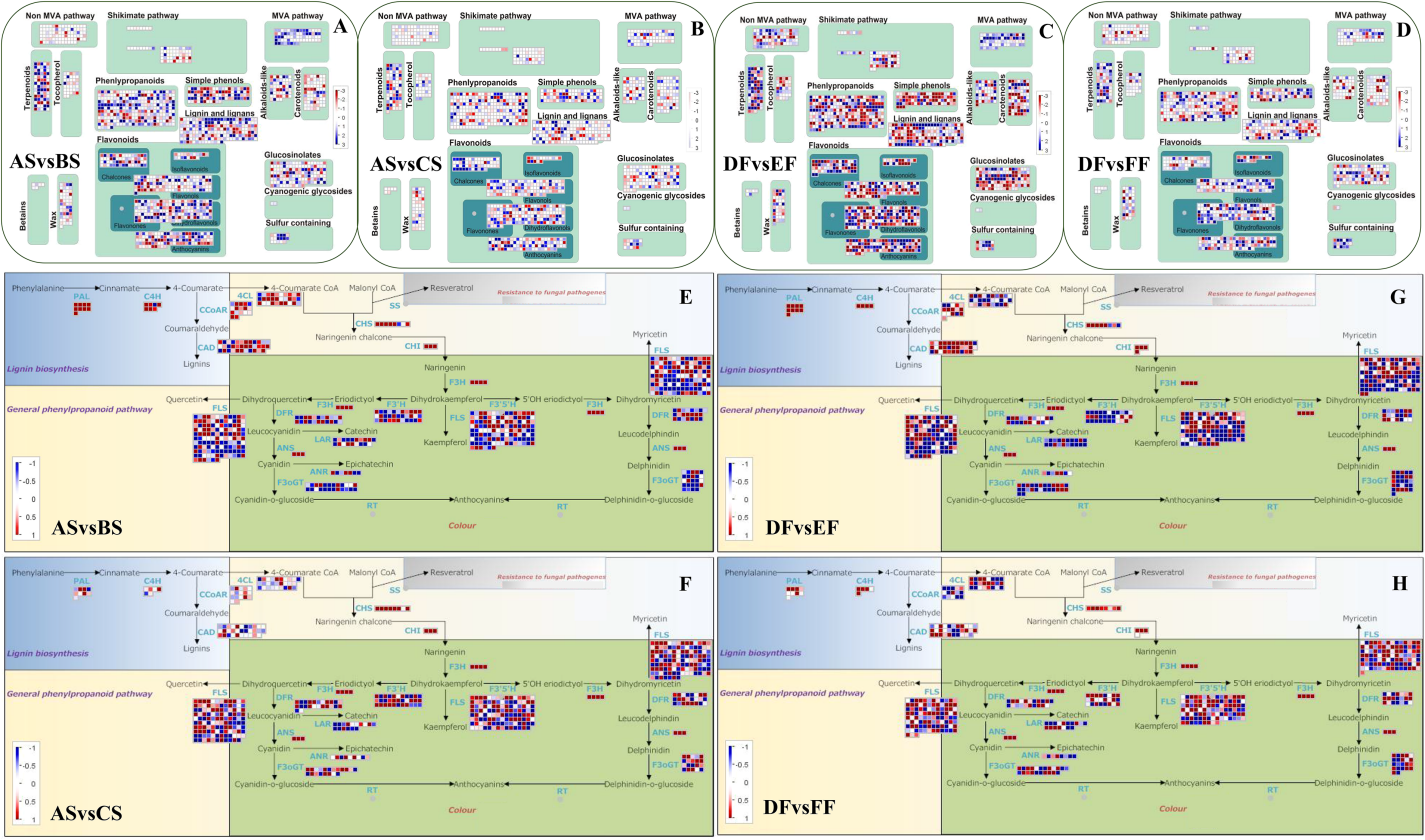
Effect of heat and cold treatment on the genes involved in general phenylpropanoid pathway and specialized metabolism in seeds and fruits of strawberry. The relative transcription levels of different genes in treated samples with heat and cold treatment compared to control were displayed in the heatmap. **(A–D)** represent the specialized metabolism in the comparison group of ASvsBS, ASvsCS, DFvsEF, and DFvsFF, respectively. The overview mainly consists of terpenoids, tocopherol, phenlypropanoids, simple phenols, lignin and lignans, flavonoids, MVA pathway, alkaloids, carotenoids, glucosinolates, and sulfur containing. Blue color represents upregulation, while red color represents downregulation. **(E–H)** represent the phenylpropanoid pathway in the comparison group of ASvsBS, ASvsCS, DFvsEF, and DFvsFF, respectively. Blue represents downregulation, while red represents upregulation. PAL, C4H, 4CL, CCoAR, CAD, CHS, FLS, DFR, ANS, F3oGT, F3H, F3’H, and F3’5’H represent phenylalanine ammonialyase, cinnamate-4-hydroxylase, 4-coumaroyl-coA synthase, cinnamoyl-CoA reductase, cinnamyl alcohol dehydrogenase, chalcone synthase, flavonol synthase, dihydroflavonol reductase, anthocyanidin synthase, flavonol-3-O-glucosyl transferase, flavanone 3-hydroxylase, flavonoid 3’-hydroxylase, and flaconoid-3’,5’-hydroxylase, respectively. Samples were divided into two groups including seeds (RT (AS), heat (BS), and cold storage (CS)) and fruits (RT (DF), heat (EF), and cold storage (FF)).

Temperature changes also had a very significant effect on the specialized metabolism pathways of fruits and seeds. Heat and cold treatments changed the transcription level of genes involved in the biosynthesis of terpenoids, phenlypropanoids, simple phenols, lignin, and lignans (**Figures 3A-D**). At the same time, heat temperature down-regulated most of the genes in the MVA pathway, and also has a significant effect on the color-related flavonoids and carotenoid pathway genes (**Figures 3A, C**). In addition, genes related to biosynthetic pathway of flavonoids were also clearly regulated in heat and cold treatments (**Figures 3E-H**). Among the responsive genes, phenylalanine ammonia-lyase (PAL), cinnamate-4-hydroxylase (C4H), chalcone isomerase (CHI), flavanone 3-hydroxylase (F3H), and anthocyanidin synthase (ANS) were the most highly downregulated, especially under heat condition. In seeds, the number of downregulated C4H and 4-coumarate: CoA ligase (4CL) genes was more than berries under heat treatment, which may be related to the increase of brown color in seed. Compared to heat treatment, postharvest cooling led to lower expression of chalcone synthase (CHS), flavonol synthase (FLS), leucoanthocyanidin reductase (LAR), flavonol 3-o-glucosyltransferase (F3oGT), and flavonoid 3′-hydroxylase (F3’H) family genes, resulting in the color difference of strawberry fruits and seeds under RT, heat, and cold conditions. In summary, heat and cold treatments affected the transcription level of transcription factor, and the genes involved in the biosynthesis of hormone, terpenoids, phenlypropanoids, flavonoids and carotenoid, resulting in the color and resistance difference of strawberry fruits and seeds under RT, heat, and cold conditions.

### Metabolic profiling of strawberry under different storage temperature modes

**Figure 4:**
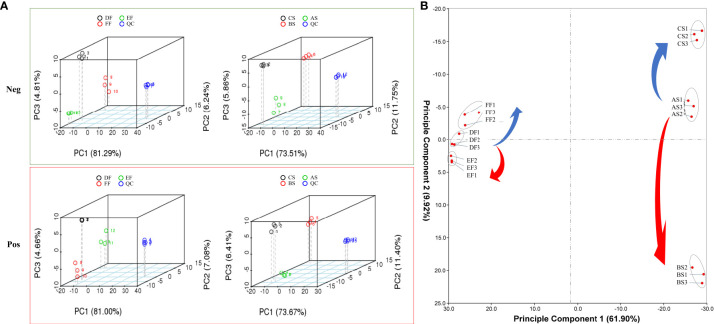
PCA for the metabolites in extracted fractions analyzed by LC-ESI-ion trap MS/MS. **(A)** PCA analysis in negative and positive ion modes. Quality Control (QC) was an equivalent standard, which is used to evaluate the stability and signal response strength of the instrument during metabolite detection. **(B)** PCA scatter plot of samples dehydrated under different temperature based on their metabolic data. The samples in the same dehydration stage were wrapped by gray solid circles. Samples were divided into two groups including seeds (RT (AS), heat (BS), and cold storage (CS)) and fruits (RT (DF), heat (EF), and cold storage (FF)).

Through database comparison, we conducted qualitative and quantitative analysis of metabolites. A total of 552 components were identified in positive ion mode and 679 in negative ion mode (**Table S5**). Principal component analysis (PCA) was used to evaluate the overall metabolic differences between samples in each group and the degree of variability between samples within the group (**Figures 4A, B**). The results showed that the repeatability of the same treatment met the requirements, and there were significant differences between different treatments. It can be seen from the scattered point distribution that the scores of seeds and fruits change regularly under different temperature modes (**Figure 4A**). The longer distance between seed and fruit in the PC1 axis (61.9%) represents the difference in the response of different organs to temperature changes while the PC2 axis is closely related to temperature changes that was 9.92% (**Figure 4B**). From RT to cold treatment, seeds and fruits all move to the negative semi-axis of PC2, and from RT to heat treatment, move to the positive semi-axis. These results represent the similarity of how seeds and fruits respond to different temperature treatments.

**Figure 5:**
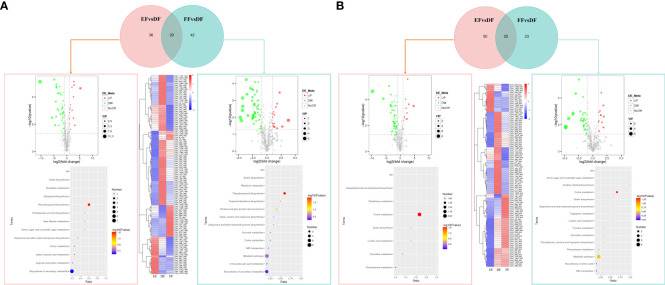
Overview of metabolic profiles in strawberries at different temperature in negative **(A)** and positive **(B)** ion modes. Venn diagram of the number of downregulated and upregulated components in different comparison group. Volcano map for the significance level of the DEGs. The horizontal axis represents the fold change of DEGs, and the vertical axis represents the significance level of the difference. Padj indicates the corrected *p* value after the multiple hypothesis test, Cluster heat map of metabolic profiles of different samples. The normalized metabolites content data after taking the logarithm was prepared for making the heatmap. KEGG enrichment pathways of differential metabolites in different comparison groups. The size of the dot represents the number of differential metabolites enriched in different pathways, and their color represents the significance level of the difference. Ratio represents the ratio of the number of differential metabolites annotated to the KEGG pathway to the total number of differential metabolites.

The detected metabolites in the positive and negative modes were analyzed by a clustered heat map. The heat and cold treatments cause significantly differences in metabolite levels (**Figures 5A, B**). The abundance of metabolites decreased in strawberry fruits were more than increased under cold and heat conditions than RT. Especially in the positive mode, there were 51 metabolites with decreased abundance under heat condition (**Table S5**). Under cold condition, the abundance of key metabolites in fruit were decreased, including aroma-related metabolites such as 4-coumaric acid, 3-coumaric acid, coumarin, coumarone, mevalonic acid, DL-malic acid, and pogostone, and amino acid-related metabolites such as arginine and L-5-hydroxytryptophan, while the malic acid metabolite 2-isopropylmalic acid was down-regulated. L-glutathione oxidized was increased under cold treatment and decreased under heat condition in seeds. DL-arginine showed decreased abundance under both heat and cold treatments in seed and fruits. Temperature changes also altered the hormone content in strawberry. Under heat condition, the content of abscisic acid (ABA), methyl jasmonate (MeJA), and gibberellin (GA) increased in seed and fruits. In the seed, cold treatment increased ABA, while heat treatment decreased ABA and hiked MeJA content. Under heat condition, the content of ABA, MeJA, and GA increased.

The metabolic pathways in fruits under heat to RT storage condition (EFvsDF) and cold to RT storage condition (FFvsDF) were enriched to 12 and 13 paths in negative ion modes and 7 and 14 paths in positive ion modes, respectively (**Table S6**). Pathways were mainly included purine and pyrimidine metabolism, glutathione metabolism, terpenoids metabolism (sesquiterpenoid, diterpenoid and triterpenoid biosynthesis, ubiquinone and other terpenoid-quinone biosynthesis), linoleic acid metabolism, alpha-linolenic acid metabolism, zeatin biosynthesis, phenylalanine metabolism, and biosynthesis of specialized metabolites (**Figure 5**). The response of seeds to temperature changes was more sensitive than that of fruits, and the number of abundance-increased metabolites was greater than decreased compounds (**Figure S5**; **Table S5**). The metabolic pathways in BSvsAS and CSvsAS were enriched to 14 and 28 pathways in negative ion modes and 9 and 13 pathways in positive ion modes, respectively (**Table S6**). In addition to the enrichment pathways of fruit under cold and heat storage temperature, the different metabolites were specifically enriched in flavonoid, flavone, and flavonol biosynthesis in seeds. Therefore, the heat and cold treatments affected lots of metabolic pathways, causing significantly differences in metabolite levels and the hormone content.

### Correlation analysis between transcriptome and metabolome

To evaluate the relationship between transcriptome and metabolome, we integrated the KEGG pathway enrichment results for DEGs and metabolome (**Figure S6**). In the positive ion mode, FFvsDF, EFvsDF, CSvsAS, and BSvsAS were enriched to 11, 6, 10, and 7 pathways, respectively, while in the negative ion mode enriched to 11, 11, 23, and 13, respectively (**Table S7**). Among significant pathways, the pathways related to metabolic activity like specialized metabolites and terpenoid biosynthesis were more enriched. Under cold condition, the difference in metabolites of fruits was mainly reflected in terpenoid backbone biosynthesis, amino acid biosynthesis and metabolism (valine, leucine, tyrosine, phenylalanine, tyrosine, tryptophan and isoleucine), and pyruvate metabolism. Meanwhile, heat condition was mainly reflected in phenylalanine metabolism, phenylpropanoid biosynthesis, glutathione metabolism, and terpenoid biosynthesis (diterpenoid, sesquiterpenoid and triterpenoid). The metabolites of the seeds were more abundant than those in fruits, including biosynthesis of amino acids (alanine, cysteine, methionine, phenylalanine, tyrosine, tryptophan, histidine, lysine, glycine, serine, threonine, and arginine), phenylpropanoid (flavonoid and phenylpropanoid biosynthesis), and terpenoid biosynthesis (sesquiterpenoid and triterpenoid, alkaloid, chlorophyll, and ubiquinone). The combined analysis of the transcriptome and metabolome confirmed that the synthesis and metabolism of amino acids and terpenoids, as well as the phenylpropane pathway, play an important role in response to temperature changes.

### Response of related genes to defense system under heat and cold treatments

**Figure 6:**
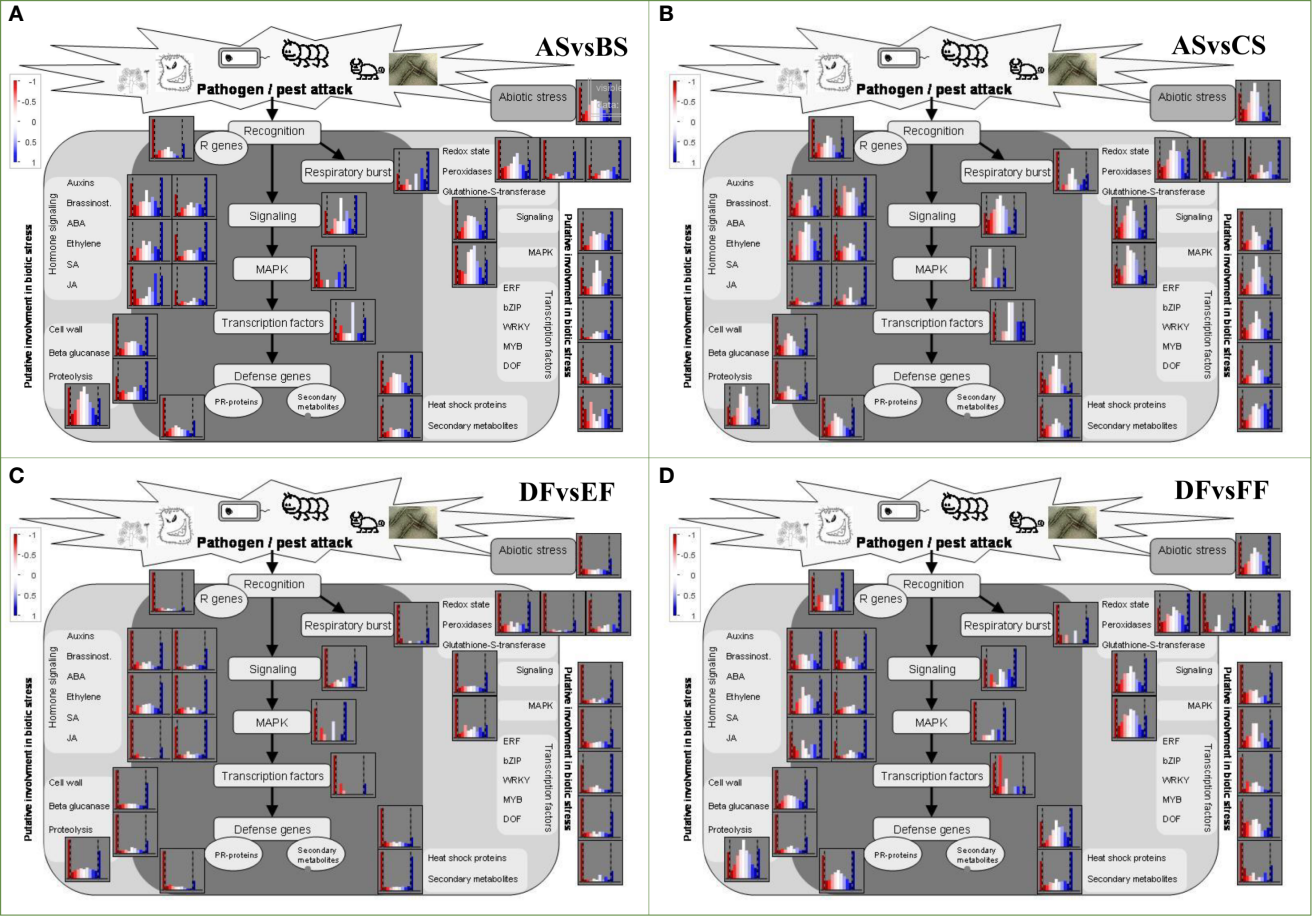
DEGs assigned to abiotic stress based on Mapman software. This map consisted of the genes that participated in abiotic stress, redox, signaling, transcription factors, heat shock proteins, pathogenesis protein, hormone relative genes, as well as the genes implicated in cell wall and proteolysis. Blue represents upregulation, while red represents downregulation. **(A–D)** represent the comparison group of ASvsBS, ASvsCS, DFvsEF, and DFvsFF, respectively.

Cell wall modification is one of the important factors in quality control of fruit and seed. On the whole, the up-regulation of cell wall metabolism-related genes under heat condition was higher than cold (**Figures 6A-D**). Four enzymes involved in cell wall metabolism including cellulase (Cx), xyloglucan endo-transglycosylase (XET), pectate lyases (PL), andα-L-Arabinofuranosidase (α-L-Af) were investigated (**Table S8**). In the seed, heat increased the number of up-regulated genes, while cold treatment down-regulated 14 of 18 genes belonged to XET genes and 4 of 7 genes belonged to Cx genes. In the fruits, the four enzyme genes were inhibited with different degrees. Both heat and cold treatments affected the content of reactive oxygen species (ROS) and scavenging system. In AS vs BS, the activity of respiratory burst NADPH oxidase (Rboh) increased while copper amine oxidase (AOC) activity was inhibited (**Table S8**) but both were down-regulated in AS vs CS. Superoxide dismutases (SODs) as a group of defensive enzymes were up-regulated in AS vs BS, AS vs CS, DF vs FF, and DF vs EF, which convert disproportionate ${\text{O}}_{2}^{-}$ into oxygen and H_2_O_2._ Furthermore, CAT and POD enzymes act as ROS scavenging and expressed in DF vs FF while were down-regulated in DF vs EF.

Among responsive genes, most HSPs were up-regulated under heat treatment, although HSP20, HSP70, and HSP90 were identified as differentially expressed genes (**Table S8**). Under cold condition, the number of down-regulated genes was more than up-regulated genes (**Figure 6**). In AS vs BS, 42 of 66 HSPs with high molecular weight (HSP70 and HSP90) and 32 of 47 HSP20 were up-regulated. In AS vs CS, 49 of 69 HSPs (18 HSP20, 19 HSP70, 12 HSP90) were down-regulated. Most of the PR-protein genes were also up-regulated under heat condition, while the number of up-regulated and down-regulated genes was basically the same under cold treatment (**Figures 6B, D**). In AS vs BS, there were 32 PR-10 genes (all up-regulated) and 13 chitinase (10 of 13 up-regulated), while 14 PR-10 genes and 3 chitinase genes increased in AS vs CS (**Table S8**).

The above results showed that defensive mechanisms could be different in seeds and fruits of strawberry when were treated with different storage temperatures like cold and heat treatments. In total, according to transcriptome data, heat treatment had a greater impact on the entire defense system of strawberries than cold treatment.

### Metabolic response of YZs-overexpressing fruits under different storage temperatures

**Figure 7:**
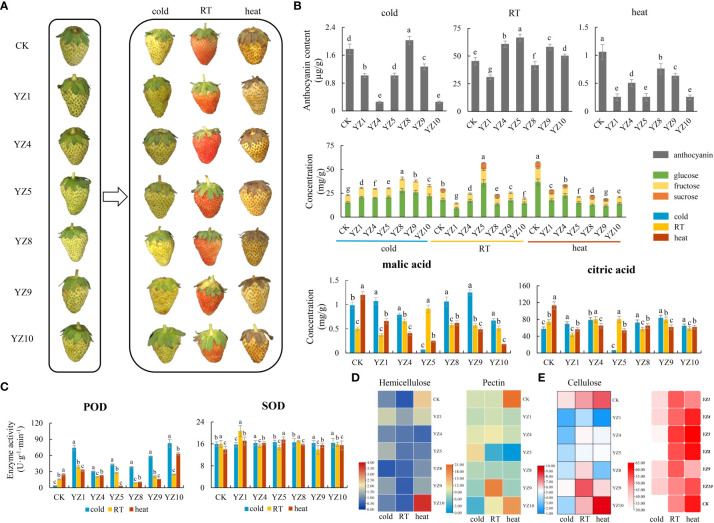
Physiological and biochemical changes in the fruits of YZs-overexpressing strawberry. **(A)** YZs were overexpressed in strawberry fruits treated with different storage temperatures like cold (4°C), heat (37°C), and room temperature (25°C). YZ1: Hsp20/alpha crystallin family; YZ4: Universal stress protein family; YZ5: Senescence regulator; YZ8: EF-hand domain pair; YZ9: Histone-like transcription factor (CBF/NF-Y) and archaeal histone; YZ10: Protein phosphatase 2C; **(B)** The changes of the content of anthocyanin, sugars (glucose, fructose, and sucrose), and acids including malic and citric acid; **(C)** The changes of the activity of SOD and POD; **(D)** Heat map analysis of the changes of the content of cellulose, hemicellulose, and pectin; **(E)** Heat map of the berry aroma.

We screened and selected six genes according to FPKM parameter (Fragments Per Kilobase Million), which calculates the gene expression levels by the number of fragments per kilo base of transcript sequence per million base pairs sequenced (**Table S1**). This parameter significantly changed under temperature treatments for six genes, especially heat treatment that may play an important role in seeds and fruits of strawberry under different storage temperatures. Therefore, we used the transient transformation system to express them in strawberries at the big green fruit stage. The results showed that both heat and cold treatments inhibited the color change of strawberries during the big green fruit period (**Figure 7A**). Transformed fruits with *YZ4, YZ5, YZ9*, and *YZ10* promoted coloring of fruits at RT but inhibited it under cold and heat treatments. Meanwhile, transformation of fruits with *YZ8* led to the promotion of strawberry fruit coloring at cold treatment but inhibited it under RT and heat conditions. In fruit transformed with *YZ1*, we observed the inhibition of anthocyanin content under RT, cold, and heat conditions (**Figure 7B**). The glucose and fructose content of strawberry fruits also increased under cold condition slightly and the sucrose content was completely degraded in transformed fruits with *YZs*. The sugar components decreased severely in transformed fruits with *YZs* under heat condition but slightly decreased under RT, except for the transformed fruits with *YZ5* that accumulated more (**Figure 7B**). In addition, heat treatment reduced the content of citric acid and malic acid in strawberries. The content of citric acid and malic acid in transformed fruits with *YZ9* increased under cold condition but decreased under heat condition. The content of both organic acids in transformed fruits with *YZ5* dropped sharply under cold and heat conditions (**Figure 7B**). The activities of POD and SOD were tested after *YZs* expressed in strawberries. There is not much change in SOD activity under cold conditions, but POD activity is greatly increased. The activity of SOD increased to a certain extent under heat condition. The activities of SOD and POD in strawberry fruits after *YZ1* and *YZ10* expressed increased under any condition (**Figure 7C**). The content of cellulose, hemicellulose, and pectin in the cell wall was the same in YZs expressed fruits, and both cold and heat treatments increased the content of hemicellulose. Under RT condition, the content of pectin and cellulose increased in transformed fruits with *YZ9*, and the content of three substances reached at the highest level at fruit transformed with *YZ10* in strawberry fruits (**Figure 7D**).

The type and content of aroma determine the flavor and individualism of the strawberry fruit. In this study, we detected the changes of the aroma components in the fruits of *YZs-*overexpressing strawberry. In total, most aroma components were detected after heat treatment (**Table S9**). The aroma components in transformed fruits with *YZ5* increased than non-transforming fruits (CK) but under RT condition, aroma components were more in the transformed fruits with *YZ1, YZ4, YZ5, YZ8*, and *YZ9* than non-transforming fruits. Meanwhile, the aroma components in transformed fruits with *YZ1, YZ4, YZ9*, and *YZ10* were significantly reduced under heat condition than non-transforming fruits (**Figure 7E**). Heat and cold reduced the content of linalool, which is the characteristic aroma component of strawberry. Under RT condition, overexpression of *YZ1, YZ4, YZ9*, and *YZ10* in fruit strawberry increased linalool content into 0.05%, 0.07%, 0.15%, and 0.07%, respectively. Meanwhile, heat treatment increased the content of 1-octanol in overexpressed fruits with *YZ1, YZ5*, and *YZ8* (**Figure S7**).

**Figure 8:**
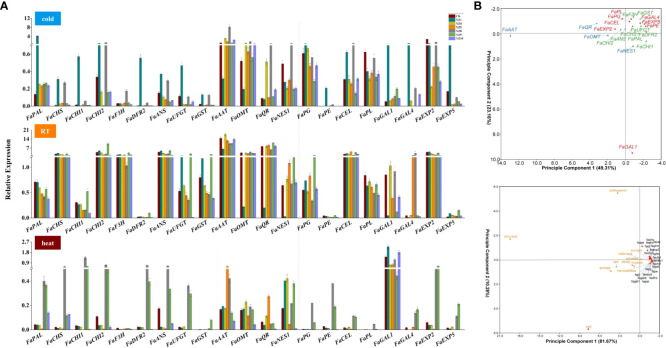
The expression level of key genes related to aroma, cell wall, and anthocyanin synthesis in the fruits of YZs-overexpressing strawberry and PCA analysis. **(A)** The expression levels of genes related to anthocyanin synthesis (phenylalanine ammonia-lyase (FaPAL), chalcone synthase (FaCHS), chalcone isomerase (FaCHI1, FaCHI2), flavanone 3-hydroxylase (FaF3H), dihydroflavonol 4-reductase (FaDFR1, FaDFR2), anthocyanidin synthase (FaANS), UDP glucose: flavonoid 3-o-glycosyl transferase (FaUFGT), glutathione s-transferase (FaGST)), aroma formation (alcohol acyltransferases (FaAAT), o-methyltransferase (FaOMT), quinone oxidoreductase (FaQR), nerolidol synthase (FaNES1)), and cell wall metabolism (polygalacturonase (FaPG), pectinmethylesterase (FaPE), endo-β-1,4-glucanases (FaCEL), pectate lyase (FaPL), β-D-galaetosidase (FaGAL1, FaGAL4), expansin (FaEXP2, FaEXP5)) under different storage temperatures; **(B)** PCA analysis of the expression level of 21 related genes, as well as other quality traits, including malic acid, citric acid, glucose, fructose, sucrose, POD, SOD, hemicellulose, pectin, cellulose, anthocyanin.

We also investigated the expression levels of genes related to aroma, cell wall, and anthocyanin synthesis the fruits of *YZs-*overexpressing strawberry. Most of these genes were highly expressed under RT condition than heat and cold treatments, and lowest under heat condition (**Figure 8A**). The expression levels of coloring-related genes including *FaPAL, FaUFGT, FaCHS, FaF3H, FaCHI1, FaCHI2, FaDFR2, FaANS*, and *FaGST* in transformed fruits with *YZ1* and *YZ8* were significantly higher than the transformed fruit with other genes under cold condition. The expression level of most coloring-related genes was relatively high in *YZ1-* and *YZ9-*overexpressed strawberry fruits under RT condition and the transformed fruits with *YZ8* and *YZ9* under heat condition. The expression of genes related to aroma increased under RT conditions than that cold and heat treatments. Meanwhile, the expression of aroma-related genes in strawberry fruits was the highest in *YZ8*-overexpressed fruits under cold condition. The expression patterns of genes related to cell wall metabolism under different temperature conditions were significantly different. The expression level of *FaGAL1* under heat treatment was significantly higher than that of cold exposure and RT, while *FaCEL* and *FaEXP2* were highly expressed in all YZs-overexpressed fruits under RT condition, which was also reflected in the principal component analysis (**Figure 8B**). Strawberry fruits had a relatively high expression level in most of related genes in *YZ1-*overexpressed fruits under cold condition, *YZ9*-overexpressed fruits under RT, and *YZ8-*overexpressed fruits under heat treatment. In terms of color and cell wall metabolism, *YA1-*overexpressed strawberry fruits were more sensitive to cold and heat conditions. In summary, the most noteworthy is that YZ1-overexpressed fruits indicated to be very sensitive to both cold and heat treatments, inhibited the color change, and increased the aroma components of strawberry. Cold treatment promoted coloring in YZ9-overexpressed fruits.

## Discussion

### Postharvest response of strawberry is dependent on the temperature fluctuation

**Figure 9:**
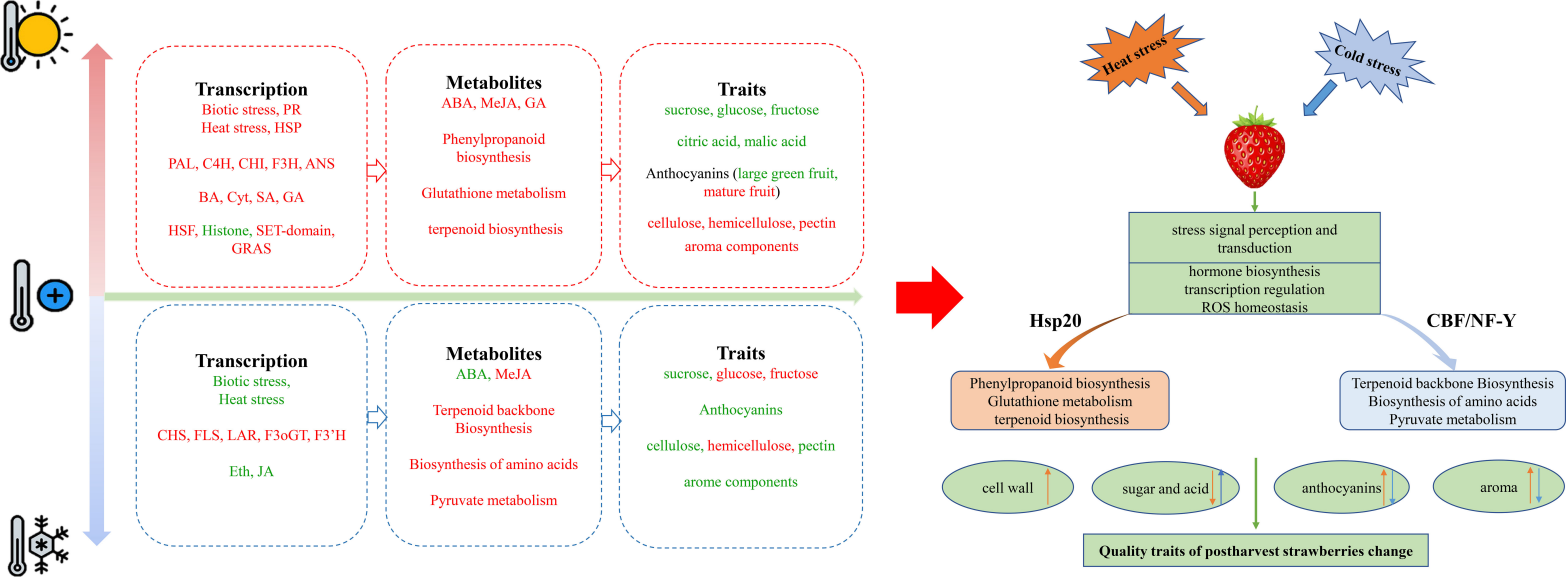
A summary of the presence of key genes, hormones, central and specialized metabolites in strawberry fruit during postharvest cooling and heat storage. Red and green represents the upregulated and down-regulated transcripts or compounds. Orange represents changes under heat stress, blue represents changes under cold stress. The up arrow represents increasing of compounds, and the down arrow represents decreasing of compounds.

Temperature changes are classified into four major groups including freezing (below 0°C), chilling (0-15°C), room temperature (25-27°C), and heat (10-15°C above ambient) (Wahid et al., 2007; Zhu et al., 2007), which have the adverse effects on metabolic and transcriptomic profiles of fruits. Strawberry flavor is formed by a mixture of numerous volatile and organoleptic compounds in ripening stage and characterize the texture and taste (Zhang et al., 2011). Temperature fluctuation also affects the content of volatile terpenes in strawberries. Postharvest cooling for 9 days significantly preserved the terpene content of ‘Akihime’ than room temperature, while degraded them in ‘Sweet Charlie’ from 15°C to 25°C (Fu et al., 2017). In total, the organoleptic and nutritional characteristics of fruits depend on the two main families of specialized metabolites including polyphenol and terpenoid compounds (Pott et al., 2019). In addition, there are a special attention in different cultivars of strawberry to determine and preserve the volatile compounds during postharvest storage. For example, the most aromatic components in ‘Benihoppe’ were linalool, nerolidol, 1-octanol, butanoic acid methyl ester, ethyl ester, hexanoic acid methyl ester, and ethyl ester (Yan et al., 2018). Furthermore, linalool and (E)-nerolidol were found to be the two major terpenoid constituents in strawberry (Ayala-Zavala et al., 2004; Liu et al., 2016). Temperature fluctuations could also alter the fluidity of cell membranes, affect the activities of enzymes, and lead to a considerable impact on cell physiology (Zhu, 2016; Gong et al., 2020). As shown in **Figure 9**, we summarized the signal transduction, substance synthesis, and the changes in the transcription and metabolic levels of strawberries in response to heat and cold treatments through the combining of transcriptome, metabolome, and transient expression. Our results indicated that heat and cold treatment regulate the central and specialized metabolic pathways such as amino acid biosynthesis, terpenoid biosynthesis, and phenylpropanoids metabolism during postharvest periods through the mechanisms involved in biotic and abiotic stresses, and hormone signal transduction processes. In this study, heat treatment increased the aroma components during postharvest storage in strawberry fruits, however heat and cold treatments reduced the proportion of linalool.

Anthocyanins are the main polyphenols that produce fruit pigmentation. The antioxidant activity of strawberries is positively correlated with the content of anthocyanin (Wang and Lin, 2000; Gu et al., 2003). Furthermore, anthocyanin as a kind of flavonoid pigment is involved in plant tolerance to abiotic stress and accumulates in the ripening stage of grapes, cherries, and strawberries (Shin et al., 2007; Carmona et al., 2017; Martinez-Romero et al., 2017). The synthesis and accumulation of postharvest anthocyanins can improve the appearance of immature strawberries. The continuous accumulation of anthocyanins after harvest causes strawberries to appear dark red, and high temperature environments could aggravate this discoloration (Steyn et al., 2009; Hu et al., 2015; Peng et al., 2017). However, it has also been reported that high-temperature cultivation could inhibit the accumulation of anthocyanins, resulting in poor coloration of strawberries, which was similar in grapes and apples (Matsushita and T, 2016; de Rosas et al., 2017). In this study, heat inhibited the accumulation of strawberry anthocyanins during the big green fruit period and deepened the coloring of strawberries after harvest, which means the effect of heat treatment on strawberry anthocyanins is dependent on the development period (**Figures 1**, **7**) (Zhang et al., 2019). also demonstrated that heat treatment did not change the concentration of anthocyanins in semi-ripe fruits. Therefore, the synthesis and degradation of anthocyanins coexist in a high temperature environment, and the content of anthocyanins depends on the balance between synthesis and degradation (Niu et al., 2017).

The nutritional value of strawberry fruits is associated with the content of specialized metabolites, organic acids, soluble sugars, and amino acids (Zhang et al., 2011). In addition, cold treatment leads to many changes in plant metabolism, such as the increase of sugars, organic acids, and amino acids (Kaplan et al., 2004; Zuther et al., 2012; Chai et al., 2019). In this study, heat treatment reduced the content of sucrose, glucose, and fructose, as well as citric and malic acid but postharvest cooling increased the content of glucose and fructose. During strawberry fruit ripening and storage, cell wall polymers composition and structure were modified and contributed to textural changes. Our results indicated that heat treatment increased the content of cell wall cellulose, hemicellulose, and pectin (**Figure 7**). Furthermore, the expression level of cell wall metabolism-related genes enhanced during postharvest heat storage (**Figure 8**) that was consistent with the results of Langer et al. (2018), which demonstrated the modification of strawberry cell wall metabolism under heat treatment, the improvement of cellulose content and semi-fiber, and monitoring strawberry firmness. We also found that the amino acid synthesis and metabolism in fruits and seeds of strawberry play the important role in the processes of strawberry coping with temperature fluctuation (**Figure 5**). Zhang et al. (2011) also confirmed the involvement of amino acid metabolism during strawberry fruit development.

### Temperature sensing pathways monitor postharvest quality in strawberry

Cold stress could quickly trigger the expression of many transcription factors, including AP2 family transcription factors CBFs, thereby activating the expression of a large number of downstream cold response (COR) genes. The expression of CBF gene is controlled by upstream transcription factors including bHLH transcription factor ICE1 (Chinnusamy et al., 2007; Shi et al., 2012; Jin et al., 2017). *CBF* genes were rapidly and dramatically induced by cold shock and orchestrate cold tolerance in plants (Liu et al., 1998; Ye et al., 2019). In this study, the transcription factor AP2 was not significantly affected, however, bHLH, MYB, WRKY, and AS2 transcription factors responded to postharvest cooling storage (**Figure S4**). We found that histone-like transcription factor (CBF/NF-Y) and archaeal histone (YZ9) promoted the coloring of strawberry fruit, increased the content of pectin and cellulose, and hiked the aroma ratio of linalool (**Figure 9**). Furthermore, the content of citric and malic acid increased in postharvest cooling storage but reduced under heat treatment in the transformed fruits with YZ9 (**Figure 7**). This confirmed the role of YZ9 in the preservation of fruit quality under cold treatment.

The effect of low temperature on plant metabolism comes from the direct inhibition of metabolic enzymes or the reorganization of gene expression (Zhu, 2016). In the transformed fruits with the overexpression of Hsp20/alpha crystallin family (YZ1), Histone-like transcription factor (CBF/NF-Y) and archaeal histone (YZ9), and Protein phosphatase 2C (YZ10), we observed the improvement of the aroma ratio of linalool in room temperature, which demonstrate the involvement of regulatory genes in aroma components (**Figure S7**). Previous studies showed that the expression of *FaQR* significantly decreased under cold treatment, while *FaQR* and *FaOMT* were up-regulated and *FaAAT*, *FaNES*, and *FaPAL1* down-regulated with increasing of temperature (Li et al., 2015; Fu et al., 2017). Similarly, in our study, the expression of aroma biosynthesis genes like *FaAAT* and *FaNES* was much higher at cold treatment than heat, meanwhile *FaQR* and *FaOMT* also showed the same pattern (**Figure 8**), which may be related to the difference in variety of strawberry.

Hormones are signaling molecules that regulate gene expression under cold stress (Jin et al., 2017; Zhang et al., 2019). Plant hormone signaling pathways could interact with the CBF pathway to regulate cold sensing (Shi et al., 2015). For example, CBFs could reduce bioactive gibberellin levels and activate by Brassinazole-resistant 1 (BZR1)/BRI1-EMS suppressor 1 (BES1), which enhanced freezing tolerance (Achard et al., 2008; Eremina et al., 2016). CBF gene expression is also repressed by Ethylene insensitive 3 (EIN3), a transcription factor that positively regulates ethylene-dependent gene expression (Shi et al., 2012). Jasmonate zim-domain protein 1/4 (JAZ1/4) proteins as repressors of the jasmonic acid (JA) signaling pathway interact with ICE1/2 to regulate CBF expression (Hu et al., 2013). Our results also confirmed that cold treatment down-regulated most of the genes involved in the ethylene and JA signaling pathways and changed the expression of BZR transcription factors (**Figures S3**, **S4**).

A sudden increase in temperature initiates the activation of heat shock response (HSR) mechanisms and induce the expression of molecular chaperones to combat the negative effects on proteins caused by stressors (Kotak et al., 2007; Gong et al., 2020). As part of the heat response, heat shock transcription factors (HSF) could act as molecular chaperones to prevent protein denaturation and rapidly induce the expression of HSPs (Zhu, 2016). Under heat stress, HSFs are released from the HSP70/HSF and HSP90/HSF complexes, bound to misfolded proteins caused by heat stress, and activate downstream signaling pathways encoding transcription factors, enzymes, and chaperone proteins (Scharf et al., 2012; Liao et al., 2016; Ohama et al., 2017). In *Arabidopsis*, HSP100, HSP90, HSP70, HSP60, and small HSPs (sHSPs) function in thermotolerance (Kotak et al., 2007). Our results indicated that HSP20, HSP70, and HSP90 play an important role in the treated strawberry with heat and cold (**Figure 6**, **Table S8**). Among them, the overexpressed strawberries with Hsp20/alpha crystallin family (YZ1) inhibited the color change of strawberries, decreased sugar and acid content, and significantly increased SOD and POD activities (**Figure 7**). Since ROS are produced soon after the onset of heat stress and function as early messengers in stress signaling pathways (Volkov et al., 2006; Banti et al., 2010), therefore, ROS homeostasis is necessary for thermotolerance by activating the ROS scavenging system. High temperature increases the accumulation of strawberry ROS (Peng et al., 2017; Zhang et al., 2019). The accumulation of ROS and the destruction of membrane structure affect postharvest browning (Lin et al., 2014). Ascorbate peroxidases (APXs) and catalases (CATs) are two types of ROS-scavenging enzymes that detoxify the ROS (Baxter et al., 2014). In this study, we confirmed the important role of antioxidant enzymes including SOD, POD, and CAT under temperature changes, indicating that temperature stress balance the ROS accumulation after strawberry harvest.

## Conclusion

We found the effect of postharvest heat storage on the color of strawberry fruits was developmental stage-dependent. In the big green fruit period, high temperature inhibits the accumulation of anthocyanin but deepens the color of strawberries after harvest. In our study, heat treatment increased the metabolic levels the content of ABA, MeJA, up-regulated HSF, SET-domain, GRAS transcription factors, and down-regulated Histone transcription factor. Cold treatment increased the content of MeJA and decreased the content of ABA and genes expression involved in biotic and heat stress. Three major metabolism pathways including terpenoid biosynthesis, amino acid biosynthesis and metabolism, and pyruvate metabolism responded to cold treatment, while phenylalanine metabolism, phenylpropanoid biosynthesis, glutathione metabolism, and terpenoid biosynthesis were activated in postharvest heat storage of fruits. *HSF20* (*YZ1*)-overexpressed fruits indicated to be very sensitive to both cold and heat treatments, inhibited the color change, and increased the aroma components of strawberry. Cold treatment promoted coloring in Histone-like transcription factor (CBF/NF-Y) and archaeal histone (YZ9)-overexpressed fruits. Therefore, we concluded that the temperature fluctuation affects postharvest responses of strawberries and links with color, flavor, and the nutritional value of fruit.

## Data availability statement

The original contributions presented in the study are publicly available. This data can be found here: NCBI, PRJNA783909.

## Author contributions

TZ, conceptualization, data curation, investigation, writing - original draft, writing - review and editing. JL, investigation, software, and writing - original draft. ES, writing - original draft, writing - review and editing. JC, conceptualization, methodology, funding acquisition. HJ, funding acquisition, methodology, supervision, writing - review and editing. All authors contributed to the article and approved the submitted version.

## Funding

This work was supported by grants from Key Project for New Agricultural Cultivar Breeding in Zhejiang Province (2021C02066-6), Jiangsu Excellent Youth Fund Project (BK20180076), National Natural Science Foundation of China (31872047), Jiangsu Independent Innovation of Agricultural Science and Technology (CX(19)3088), Fundamental Research Funds for Central Universities (KYXJ202003).

## Acknowledgments

We thank Ruiping Tian from the State Key Laboratory of Crop Genetics and Germplasm Enhancement for helping analyze GC-MS data.

## Conflict of interest

The authors declare that the research was conducted in the absence of any commercial or financial relationships that could be construed as a potential conflict of interest.

## Publisher’s note

All claims expressed in this article are solely those of the authors and do not necessarily represent those of their affiliated organizations, or those of the publisher, the editors and the reviewers. Any product that may be evaluated in this article, or claim that may be made by its manufacturer, is not guaranteed or endorsed by the publisher.
