# Supplementary material for: Transcriptomic and metabolomic profiling of strawberry during postharvest cooling and heat storage
Source: Front Plant Sci. 2022 Oct 12;13:1009747. doi: 10.3389/fpls.2022.1009747 (PMC9597325; doi:10.3389/fpls.2022.1009747)
Supplement: Supplementary file 1 [file DataSheet_1.docx]

**Supporting Information**

**Transcriptomic and metabolomic profiling of strawberry during postharvest cooling and heat storage**

Ting Zheng^1,2#^, Jinhua Lv^2#^, Ehsan Sadeghnezhad^2^, Jianhui Cheng^1*^, Haifeng Jia^2^*

^1^ Institute of Horticulture, Zhejiang Academy of Agricultural Sciences, Hangzhou, 310021, China

^2^ College of Horticulture, Nanjing Agricultural University, Nanjing 210095, China

*Corresponding author.

*Email addresses*: [jiahaifeng@njau.edu.cn](mailto:jiahaifeng@njau.edu.cn) (H. Jia), chengjianhui@zaas.ac.cn (J Cheng).

**Fig. S1**

**
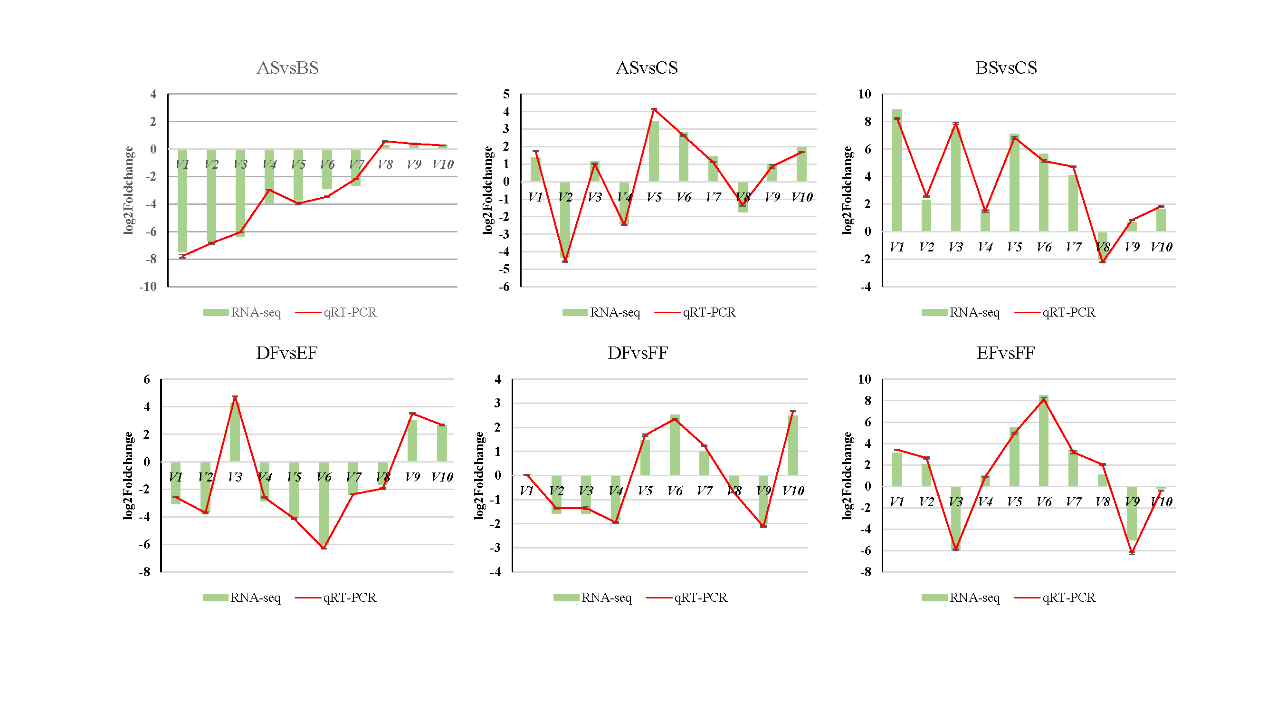
**

**Fig. S1.** Comparison of the gene transcription level detected by RNA-sequencing and qRT-PCR. The green columns indicated the sequencing result, and the red line indicates the qRT-PCR result. The Y-axis is log2 (fold change). Fold change is the ratio of expression between comparison groups. Bars represent standard deviations of the means. Samples were divided into two groups including seeds (RT (AS), heat (BS), and cold storage (CS)) and fruits (RT (DF), heat (EF), and cold storage (FF)).

**Fig. S2**


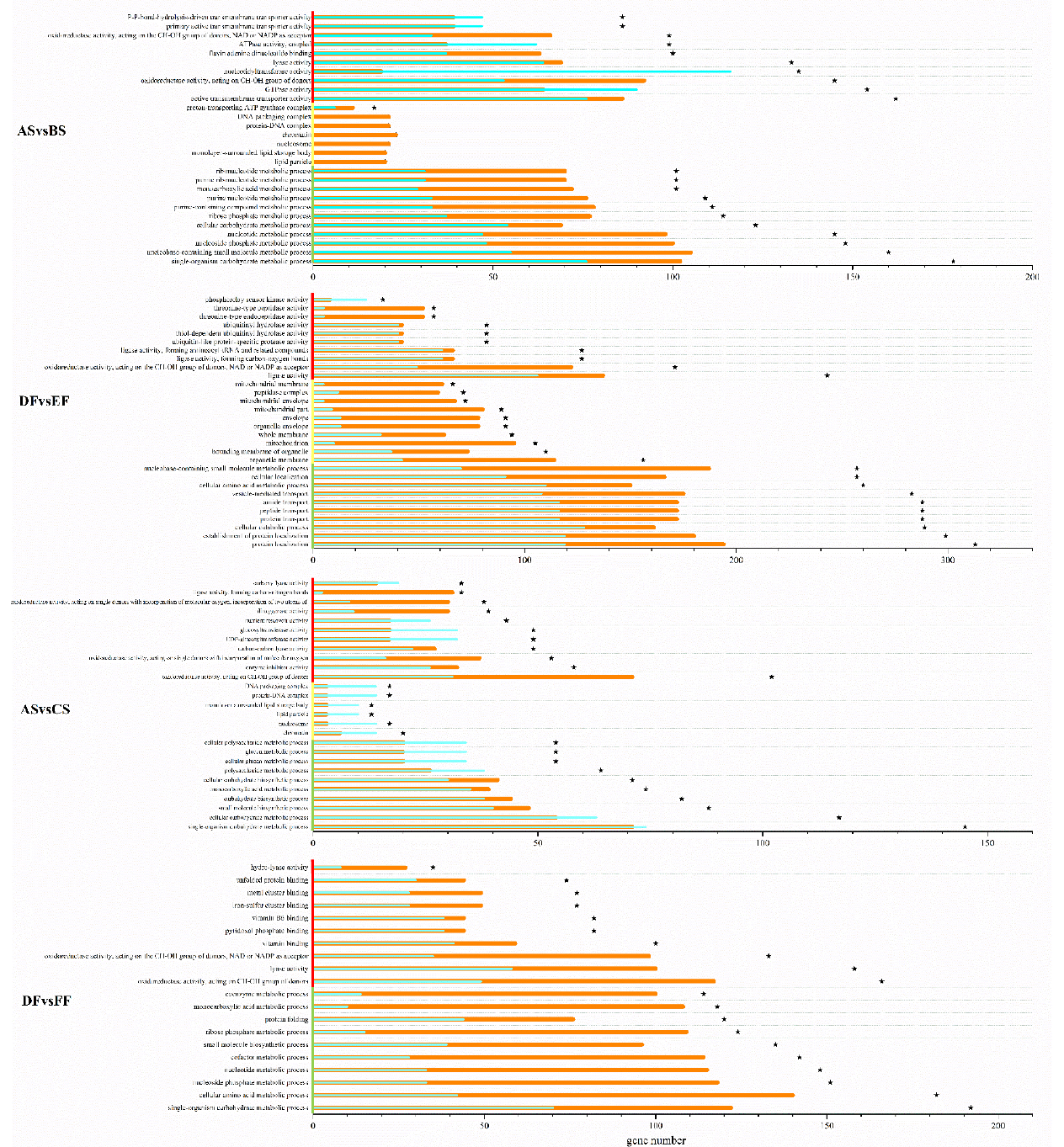


**Fig. S2.** The GO enrichment of DEGs in treated seeds and fruits with cold (4℃), heat (37℃), and room temperature (25℃). Abscissas represent the number of DEGs, and ordinate represents GO terms. Different colors were used to distinguish biological processes (BP-green), cellular components (CC-yellow), and molecular functions (MF-red). The asterisk represents the total number of DEGs enriched on the term. The orange column represents the number of up-regulated DEGs associated with the term, and the blue represents the number of down-regulated genes. Samples were divided into two groups including seeds (RT (AS), heat (BS), and cold storage (CS)) and fruits (RT (DF), heat (EF), and cold storage (FF)).

**Fig. S3**


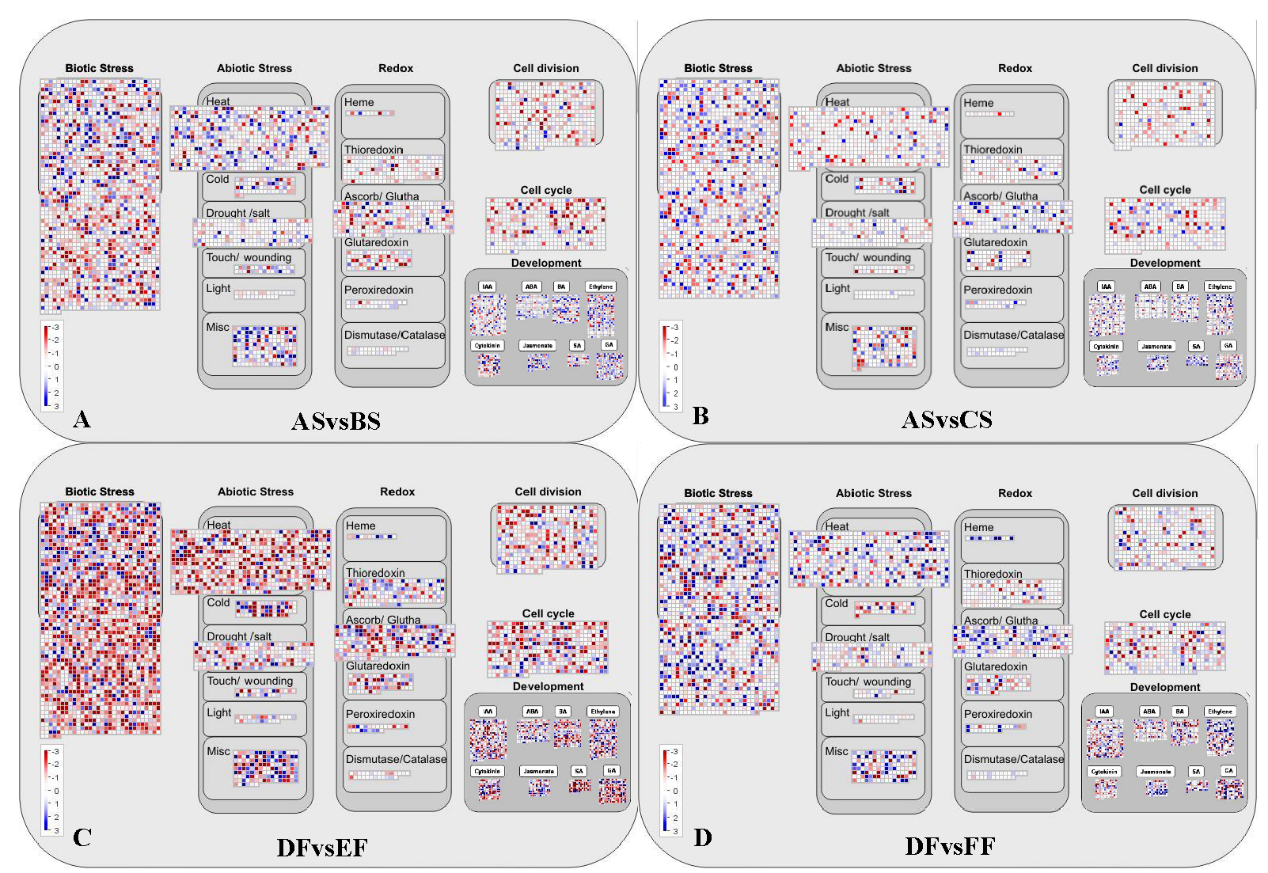


**Fig. S3.** DEGs assigned to the regulatory pathways in seeds and fruits of strawberry based on MapMan. The overview mainly consists of biotic stress, abiotic stress, redox, cell division, cell cycle, and hormone synthesis. The relative transcription levels of genes related to each part are displayed in the heatmap. Blue represents upregulation, while red represents downregulation. A, B, C, and D represent the comparison group of ASvsBS, ASvsCS, DFvsEF, and DFvsFF, respectively. Samples were divided into two groups including seeds (RT (AS), heat (BS), and cold storage (CS)) and fruits (RT (DF), heat (EF), and cold storage (FF)).

**Fig. S4**


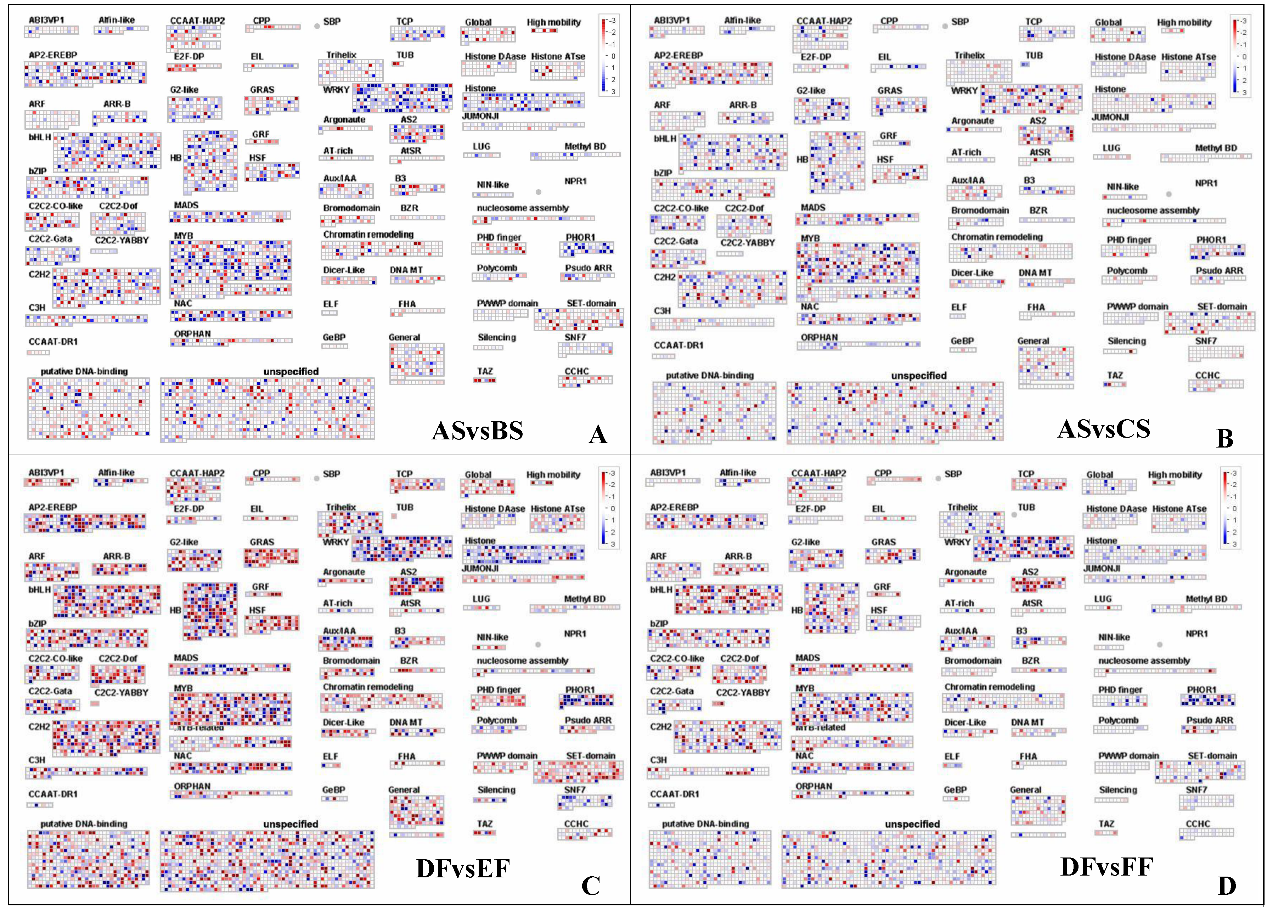


**Fig. S4.** Relative transcription level of transcription factors belonged to different families in seeds and fruits of strawberry. The main transcription factor families were listed in MapMan. Blue color represents upregulation, while red color represents downregulation. A, B, C, and D represent the comparison group of ASvsBS, ASvsCS, DFvsEF, and DFvsFF respectively. Samples were divided into two groups including seeds (RT (AS), heat (BS), and cold storage (CS)) and fruits (RT (DF), heat (EF), and cold storage (FF)).

**Fig. S5**


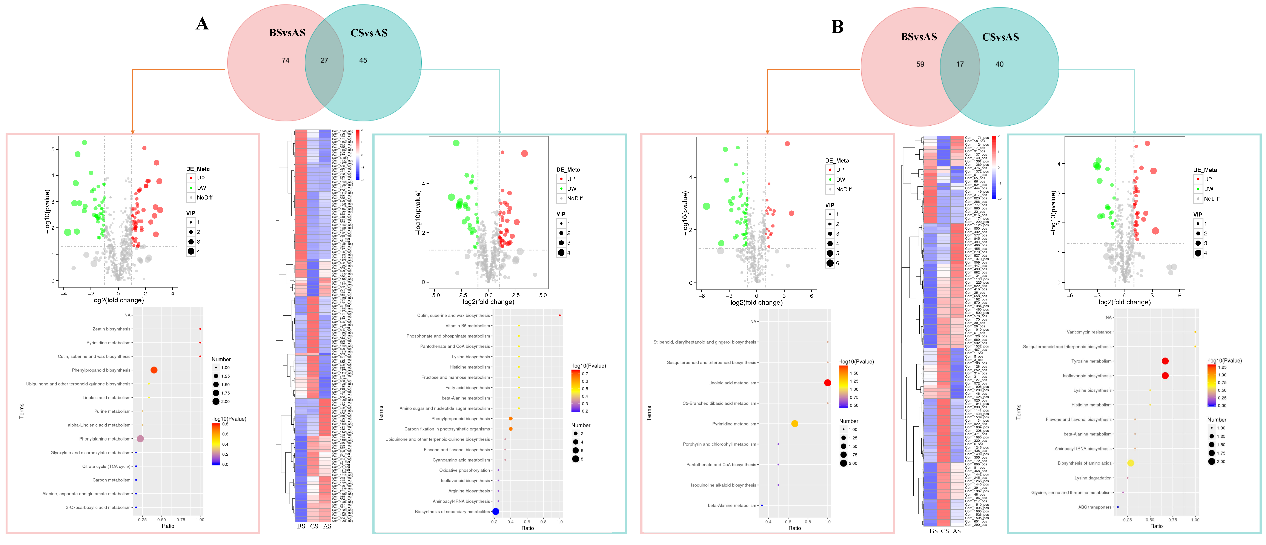


**Fig. S5.** Overview of metabolic profiles in strawberry seeds at different temperature in negative (A) and positive (B) ion modes. Venn diagram of the number of downregulated and upregulated components in different comparison group. Volcano map for the significance level of the DEGs. The horizontal axis represents the fold change of DEGs, and the vertical axis represents the significance level of the difference. Padj indicates the corrected *p* value after the multiple hypothesis test, Cluster heat map of metabolic profiles of different samples. The normalized metabolites content data after taking the logarithm was prepared for making the heatmap. KEGG enrichment pathways of differential metabolites in different comparison groups. The size of the dot represents the number of differential metabolites enriched in different pathways, and their color represents the significance level of the difference. Ratio represents the ratio of the number of differential metabolites annotated to the KEGG pathway to the total number of differential metabolites.

**Fig. S6**

**
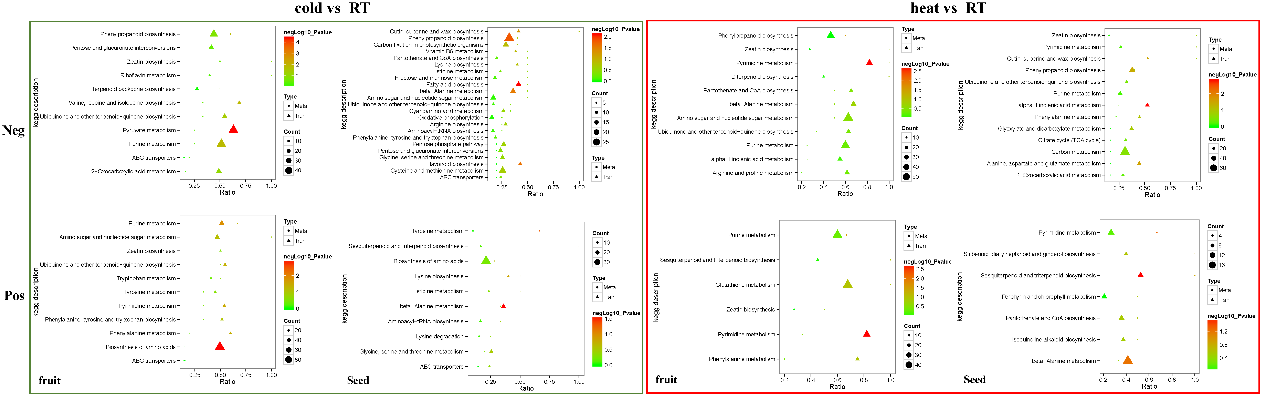
**

**Fig. S6**. Scatter plot of KEGG pathway enrichment for integrated transcriptome and metabolome. The horizontal axis value is the enrichment rate, and the vertical axis is pathway terms. Samples were divided into two groups including seeds (RT (AS), heat (BS), and cold storage (CS)) and fruits (RT (DF), heat (EF), and cold storage (FF)).

**Fig. S7**


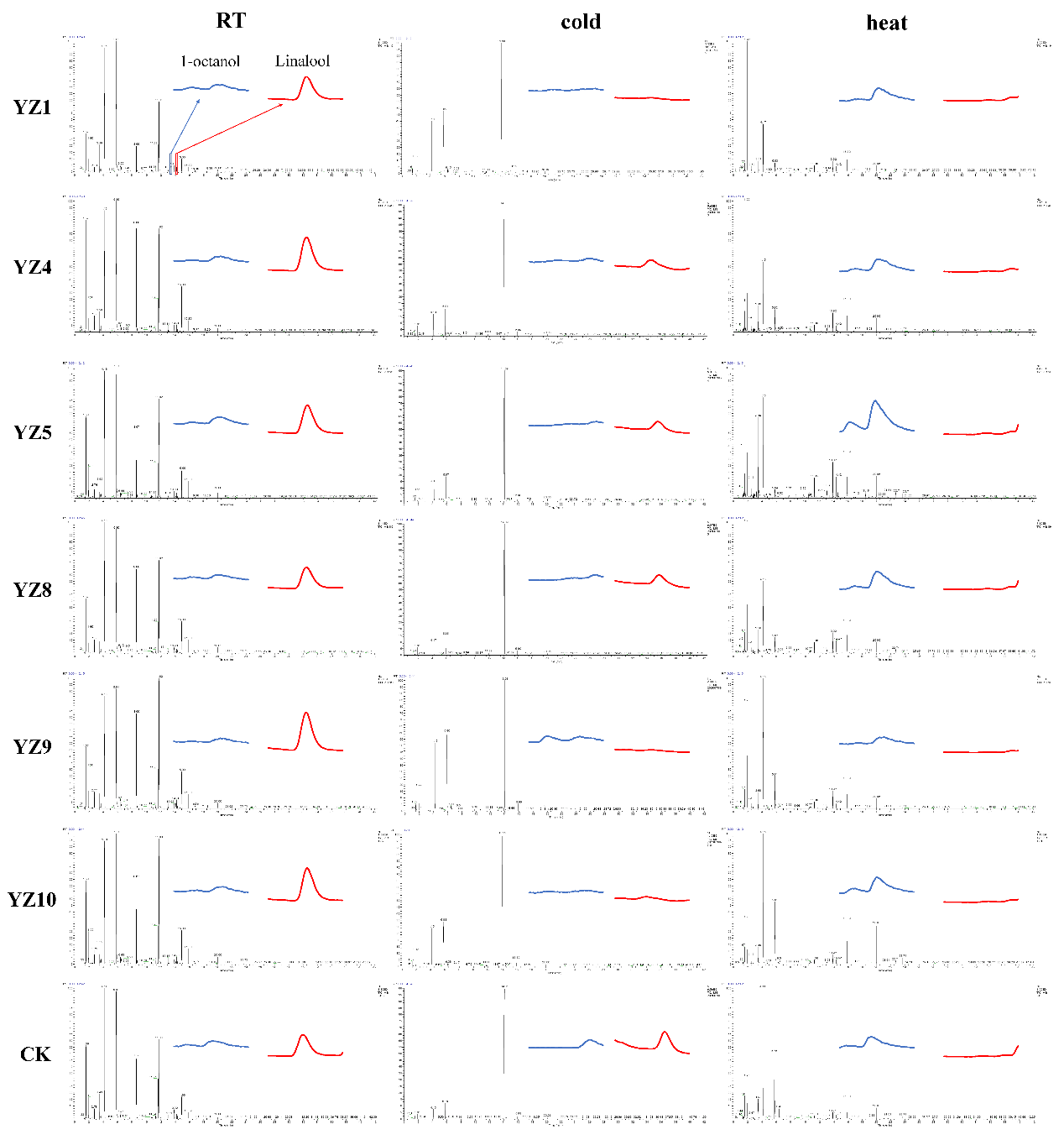


**Fig. S7** Impact of the overexpression of *YZs* on the fruit aroma components in strawberries. The red arrow means Linalool, and green means 1-octanol.
